# Significance of LDL and HDL subclasses characterization in the assessment of risk for colorectal cancer development

**DOI:** 10.11613/BM.2018.030713

**Published:** 2018-10-15

**Authors:** Milica Stevanovic, Jelena Vekic, Natasa Bogavac-Stanojevic, Jelena Janac, Zeljka Stjepanovic, Dejan Zeljkovic, Bratislav Trifunovic, Vesna Spasojevic-Kalimanovska, Aleksandra Zeljkovic

**Affiliations:** 1 Department of Medical Biochemistry, Faculty of Pharmacy, University of Belgrade, Belgrade, Serbia; 2 Medigroup General Hospital, Belgrade, Serbia; 3 Clinic of General Surgery, Military Medical Academy, Belgrade, Serbia; 4 Faculty of Medicine of the Military Medical Academy, University of Defence, Belgrade, Serbia

**Keywords:** colorectal cancer, risk prediction, lipoprotein subclasses, lipoprotein size, cost-effectiveness

## Abstract

**Introduction:**

Dyslipidaemia contributes to the occurrence of colorectal cancer (CRC). We hypothesized that qualitative changes of lipoproteins are associated with the risk for CRC development. This study analyses low-density lipoprotein (LDL) and high-density lipoprotein (HDL) diameters, as well as distribution of LDL and HDL subclasses in patients with CRC, with an aim to determine whether advanced lipid testing might be useful in predicting the risk for the onset of this malignancy.

**Materials and methods:**

This case-control study included 84 patients with newly diagnosed CRC and 92 controls. Gradient gel electrophoresis was applied for separation of lipoprotein subclasses and for LDL and HDL diameters determination. Lipid parameters were measured using routine enzymatic methods.

**Results:**

Total cholesterol, HDL and LDL-cholesterol were significantly lower in CRC patients compared to controls (4.47 mmol/L *vs*. 5.63 mmol/L; 0.99 mmol/L *vs*. 1.27 mmol/L; 2.90 mmol/L *vs*. 3.66 mmol/L; P < 0.001, respectively). Patients had significantly smaller LDL (25.14 nm *vs*. 26.92 nm; P < 0.001) and HDL diameters (8.76 nm *vs*. 10.17 nm; P < 0.001) and greater proportion of small, dense LDL particles (54.0% *vs*. 52.9%; P = 0.044) than controls. Decreased LDL and HDL diameters were independent predictors of CRC (OR = 0.5, P = 0.001 and OR = 0.5, P = 0.008, respectively), and alongside with age and HDL-cholesterol concentrations formed the optimal cost-effective model, providing adequate discriminative abilities for CRC (AUC = 0.89) and correct patients classification (81%).

**Conclusions:**

Patients with CRC have decreased LDL and HDL diameters and increased proportion of smaller particles. LDL and HDL diameters determination could be useful in assessing the risk for CRC development.

## Introduction

Colorectal cancer (CRC) is one of the most prevalent malignancies and one of the leading causes of cancer related death worldwide. Epidemiological data shows that more than a half of all cases occur in developed countries (*1*).

The precise cause of CRC is still undetermined considering that there are numerous genetic and environmental factors that may contribute to the development of cancer (*2*). In recent years, it has been documented that lipid disequilibrium is one of the main risk factors for CRC, and also for various types of cancer such as gastric, prostate, liver, lung, breast, endometrial, head and neck, and hematopoietic cancers (*3*-*5*). It has been shown that dyslipidaemia may contribute to the occurrence of CRC, probably through the interaction with process of inflammation, oxidative stress, and insulin resistance (*3*, *6*, *7*). However, previous studies have shown inconsistent results regarding the relationship between altered serum lipid profile and the onset of CRC (*8*, *9*). A possible reason could be that the above-mentioned studies were based mainly on quantitative determination of blood lipid parameters, rather than on determination of qualitative characteristics of lipoproteins. Since the studies associating lipoprotein subclasses profile and cancer are still scarce, the actual link with CRC has not yet been established. However, knowing the quality of lipoprotein particles essentially determines their functional properties, the assessment of qualitative characteristics might be extremely important for elucidating the role of lipoproteins in development of malignant diseases.

We assumed that qualitative alterations of lipoproteins are associated with the risk for CRC development. Therefore, our study was aimed to determine low-density lipoprotein (LDL) and high-density lipoprotein (HDL) particle diameters and the distributions of LDL and HDL subclasses in patients with CRC. In addition, we sought to determine if any of estimated characteristics of lipoproteins might be an independent predictor of CRC. Finally, based on the obtained results, we formulated a cost-effective model that could be used in routine clinical practice for assessing the risk of CRC development and accurate classification of CRC patients.

## Materials and Methods

### Subjects

This research was designed as a case-control study. A group of 126 patients has been recruited. All of them were admitted to the Clinic for General Surgery, Military Medical Academy, Belgrade, Serbia, between 2014 and 2016, and were all subjected to elective resection. All patients met the following eligible criteria: adult age, the first occurrence of the disease, absence of any other malignant diseases, no prior treatment with neoadjuvant therapy, no serious physical disabilities, and no use of any lipid lowering therapy. All of the 126 patients were subjected to postoperative histopathological diagnosis which confirmed the presence of adenocarcinoma in 121 patients. Furthermore, 37 patients were excluded due to incomplete clinical data or incomplete laboratory analysis, leaving 84 patients with diagnosed CRC in our study group (42 in stage B, 33 in stage C and 9 patients in stage D of CRC).

**Figure 1:**
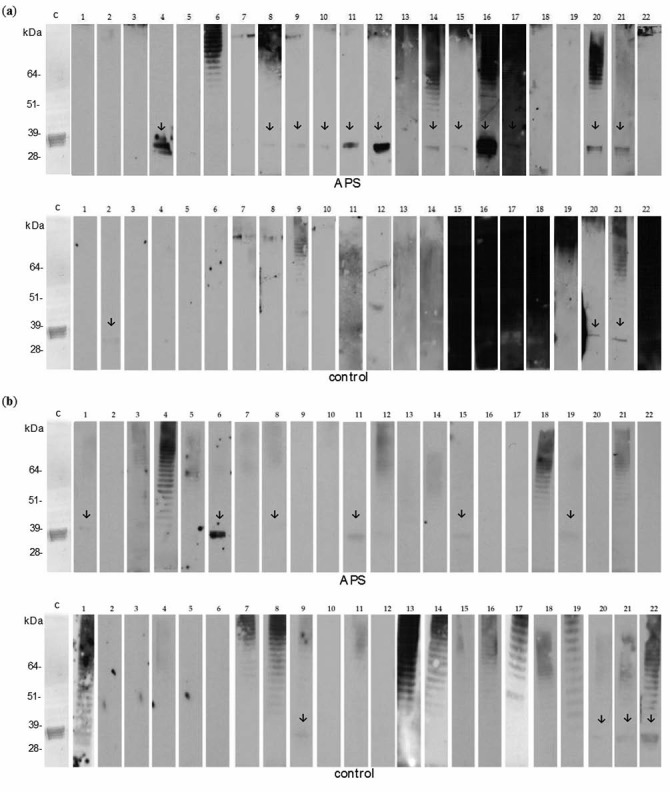
The flowchart illustrating the study cohort and approach

The control group consisted of 92 healthy adult volunteers who were subjected to general health check at General Hospital Medigroup in Belgrade, Serbia. The criteria for inclusion of volunteers to the control group were: no present malignant diseases, no past malignant diseases, no known chronic heart, kidney and liver diseases and not being subjected to any of the lipid lowering medications. The flowchart illustrating the study cohort and approach is presented in Figure 1.

Data on age, height, weight and lifestyle habits (physical activity, alcohol consumption and smoking) were collected using a questionnaire, which was designed at our Department and reviewed, adjusted and approved by the scientific and ethical boards of the Military Medical Academy. The same data collection procedures were used for patients and controls and both groups were studied in the same time period. All participants involved in the study were informed about the protocol and aim of the study. All of them signed informed consent prior to their inclusion in the study. The informed consent forms are received and archived by authors. The whole study was performed according to the Helsinki Declaration. The experimental protocol was accepted by the local ethics board for medical research.

### Methods

The patients’ blood samples were obtained before surgical procedure after overnight fasting. The control subjects’ blood samples were drawn at the commencement of medical examination also after overnight fasting. Blood was collected in BD vacutainer serum tubes with silica clot activator for obtaining the serum and BD Vacutainer K_2_EDTA tubes for obtaining the plasma samples (BD Diagnostics, Plymouth, United Kingdom). For obtaining the serum, the samples were allowed to clot 60 minutes prior to centrifugation. According to the manufacturer recommendation, the samples were centrifuged for 10 minutes at 1300xg (Eppendorf Centrifuge 5702, Eppendorf AG, Hamburg, Germany). After serum and plasma separation, the samples were aliquoted in test tubes (Ratiolab GmbH, Dreieich, Germany), frozen at - 80 °C and thawed immediately before the analyses. Lipid parameters were determined from the aliquot of serum (500 μL), while LDL and HDL subclasses from the aliquot of EDTA plasma (50 μL). Total cholesterol, triglycerides, HDL-cholesterol, LDL-cholesterol and glucose were determined by standard enzymatic methods on ILAB 300+ (Instrumentation Laboratory, Milan, Italy).

Determination of lipoprotein subclasses distribution was performed by an adapted protocol of Rainwater *et al.* A precise description of this procedure has been published previously (*10*). Shortly, separation of LDL and HDL subclasses was achieved by polyacrylamide gradient gel electrophoresis at 8 °C in a Hoefer SE 600 Ruby unit (Amersham Pharmacia Biotech, Vienna, Austria). For calibration of gels we used high molecular weight protein standards (Amersham Pharmacia Biotech, Vienna, Austria), carboxylated polystyrene microspheres (Duke Scientific, Palo Alto, USA) and standardized human samples. Following electrophoresis, the gels were stained with CBB G-250 dye for proteins (Sigma, St. Louis, USA), while with SBB dye for lipids (Sigma, St. Louis, USA). Analysis of separated fractions was performed by Image Scanner (Amersham Pharmacia Biotech, Vienna, Austria) with Image Quant software (version 5.2; 1999; Molecular Dynamics). The calculated diameters of the most prominent peaks in both lipoprotein regions were termed as dominant LDL or HDL particle diameters. We assessed relative proportions of four LDL and five HDL subclasses according to the areas of densitometric scans corresponding to particular subclass. The percentages of small, dense LDL (sdLDL) and small-sized HDL particles were determined by summing up the areas of the densitometric scan ≤ 25.5 nm and the areas ≤ 8.8 nm, respectively.

### Statistical analysis

Normality of distributions of the examined variables was tested by the Kolmogorov-Smirnov test. The results are presented as mean (X) ± standard deviation (SD) for normally distributed variables, or as median (interquartile range) for skewed variables. We used Student’s *t*-test for comparison of normally distributed continuous variables between the groups, or Mann-Whitney *U*-test for comparison of variables with skewed distributions. Categorical variables were compared by the Chi-square test of homogeneity. Using univariate logistic regression analysis, we examined the probability of CRC development. Variables that were significant in univariate analysis were entered into multivariate logistic regression analysis with forward stepwise selection aiming to find the model that best predicts the probability of CRC. Regression equation calculated for model was used for calculation of expected probabilities of CRC development (Y = 1) for a given value of predictor variables (x_1_, x_2_,…, x_n_). Expected probabilities of CRC were calculated according the equation: log (p/(1-p)) = b_0_ + b_1_x_1_ + b_2_x_2_ + … + b_n_x_n_. Expected probability of 0.5 was used as a cut-off value for classification of subjects in high or low probability group for CRC development. Accordingly, we calculated percent of correct patient classification. The variability explained by the model was assessed by the Nagelkerke R^2^. The models’ goodness of fit was examined by the Hosmer-Lemeshow test. To ascertain the strongest predictors of CRC development, we assessed the predictive ability of model by performing c-statistic (value of 1.0 was indicative for optimal discrimination and 0.5 was indicative for poor discrimination) (*11*). In order to formulate a cost-effective model, we analysed models which included only one of the parameters that are significant indicators of LDL particle size heterogeneity in addition to other significant predictors of CRC development. Accuracy and correct patient classification of cost-effective models were compared to basic model. For each odds ratio (OR) and area under the curve (AUC), we estimated the 95% confidence interval (CI). Two tailed P ≤ 0.05 was considered significant. For statistical analysis, we used PASW® Statistic v.22 software (Chicago, Illinois, USA).

## Results

Clinical and biochemical data for studied populations are shown in Table 1. CRC patients were older and prevalence of males was higher in this group. Only minority of patients declared themselves as regularly physically active. The concentrations of total cholesterol, HDL-cholesterol and LDL-cholesterol were significantly lower in the patients group compared to controls.

**Table 1:** Demographic and laboratory data of study groups

	**Patients (N = 84)**	**Controls (N = 92)**	**P**
**Age, years**	65 (32 - 83)	54 (36 - 75)	< 0.001
**Male, N (proportion)**	56 (0.67)	46 (0.50)	0.031
**BMI (kg/m^2^)**	24.6 ± 2.6	25.3 ± 2.8	0.536
**Current smoking, N (proportion)**	17 (0.20)	26 (0.28)	0.171
**Alcohol-occasional consumption, N (proportion)**	40 (0.48)	43 (0.47)	0.631
**Physical activity, N (proportion)**	10 (0.12)	76 (0.83)	< 0.001
**Glucose (mmol/L)**	5.6 (4.7 - 6.4)	5.4 (4.9 - 5.9)	0.202
**Triglycerides (mmol/L)**	1.3 (1.0 - 1.6)	1.3 (0.9 - 1.6)	0.534
**Total cholesterol (mmol/L)**	4.5 (4.0 - 5.4)	5.6 (5.0 - 6.4)	< 0.001
**HDL-cholesterol (mmol/L)**	1.0 (0.8 - 1.2)	1.3 (1.1 - 1.5)	< 0.001
**LDL-cholesterol (mmol/L)**	2.9 (2.3 - 3.5)	3.7 (2.9 - 4.3)	< 0.001
Values are expressed as median values and interquartile range, arithmetic mean ± standard deviation, or as proportions. For age, data are presented as median and range (min-max) values. Differences between groups were tested using the Mann-Whitney *U*-test for continuous variables with skewed distribution, the Chi-square test and the Student’s *t*-test for continuous variables. BMI - body mass index. HDL - high-density lipoprotein. LDL - low-density lipoprotein. P < 0.05 was considered statistically significant.			

Table 2 shows dominant diameters and distribution of LDL and HDL subclasses in the CRC patients and controls. Relative proportions of smaller LDL III subclasses were higher in patients, but the proportions of LDL I particles were greater in controls. In addition, we found elevated proportion of smaller HDL 3b and reduced proportion of larger HDL 2b particles in CRC patients.

**Table 2:** LDL and HDL dominant diameters and subclasses distribution in study groups

	**Patients (N = 84)**	**Controls (N = 92)**	**P**
Dominant LDL particle diameter (nm)	25.1 (24.1 - 26.2)	26.9 (25.8 - 27.7)	< 0.001
LDL I (%)	19 (14 - 23)	21 (17 - 30)	< 0.001
LDL II (%)	26 (23 - 31)	26 (23 - 28)	0.175
LDL III (%)	24 (21 - 27)	21 (18 - 23)	< 0.001
LDL IV (%)	29 (25 - 33)	30 (22 - 35)	0.857
sdLDL (%)	54 (48 - 59)	53 (39 - 58)	0.044
Dominant HDL particle diameter (nm)	8.8 (8.5 - 9.9)	10.2 (8.6 - 10.6)	< 0.001
HDL 2b (%)	36 (32 - 41)	39 (34 - 47)	0.013
HDL 2a (%)	23 (21 - 24)	22 (20 - 25)	0.337
HDL 3a (%)	19 (17 - 21)	19 (16 - 22)	0.815
HDL 3b (%)	11 (9 - 13)	9 (7 - 12)	< 0.001
HDL 3c (%)	10 (7 - 13)	9 (6 - 11)	0.093
Small-sized HDL (%)	41 (35 - 46)	38 (30 - 43)	0.067
LDL - low-density lipoprotein. HDL - high-density lipoprotein. sdLDL - small, dense LDL particles. Values are expressed as median values and interquartile range. Differences between groups were tested using the Mann-Whitney *U*-test. P < 0.05 was considered statistically significant.			

Increased proportions of sdLDL, LDL III and HDL 3b subclasses emerged as significant predictors of CRC. Decreased total cholesterol, LDL-cholesterol and HDL-cholesterol concentrations, smaller dominant LDL and HDL particle diameters, and reduced proportion of larger LDL I particles were significant predictors of CRC. Older age and male gender also predict CRC development (Table 3).

**Table 3:** Univariate logistic regression analysis for associations between examined variables and development of CRC

	**Unadjusted OR (95% CI)**	**P**
Age (years)	1.1 (1.1 - 1.2)	< 0.001
Male gender (%)	2.0 (1.1 - 3.6)	0.032
BMI (kg/m^2^)	0.9 (0.8 - 1.0)	0.095
Glucose (mmol/L)	1.4 (1.0 - 1.9)	0.053
Triglycerides (mmol/L)	1.2 (0.7 - 2.1)	0.497
Total cholesterol (mmol/L)	0.4 (0.3 - 0.6)	< 0.001
HDL-cholesterol (mmol/L)	0.1 (0.1 - 0.4)	< 0.001
LDL-cholesterol (mmol/L)	0.5 (0.3 - 0.7)	< 0.001
Dominant LDL particle diameter (nm)	0.5 (0.4 - 0.6)	< 0.001
LDL I (%)	0.9 (0.9 - 1.0)	< 0.001
LDL II (%)	1.0 (1.0 - 1.1)	0.193
LDL III (%)	1.2 (1.1 - 1.3)	< 0.001
LDL IV (%)	1.0 (1.0 - 1.1)	0.419
sdLDL (%)	1.0 (1.0 - 1.1)	0.004
Dominant HDL particle diameter (nm)	0.5 (0.4 - 0.7)	< 0.001
HDL 2a (%)	1.0 (0.9 - 1.1)	0.598
HDL 2b (%)	1.0 (0.9 - 1.0)	0.095
HDL 3a (%)	1.0 (0.9 - 1.1)	0.585
HDL 3b (%)	1.2 (1.1 - 1.3)	0.002
HDL 3c (%)	1.0 (1.0 - 1.1)	0.238
Small-sized HDL particles (%)	1.0 (1.0 - 1.1)	0.167
BMI - body mass index. HDL - high-density lipoprotein. LDL - low-density lipoprotein. sdLDL - small, dense LDL particles. OR - odds ratio. CI - confidence interval. P < 0.05 was considered statistically significant.		

Since a single biomarker is unlikely to provide accurate information on the risk for CRC development, we formulated the model that has incremental value in CRC screening (Table 4). Six variables (age, HDL-cholesterol, dominant HDL and LDL diameters, relative proportions of sdLDL and LDL III subclasses) were selected as independent predictors of CRC. A good fit was achieved (Hosmer-Lemeshow χ^2^ = 13.189, d.f. = 8, P = 0.105) and model explained 62% of the variation in the dependent variable (Nagelkerke R^2^ = 0.618). The resulting AUC was 0.91 (0.86 - 0.95), P < 0.001 with 81% correct classification of patients.

**Table 4:** Independent predictors of CRC selected by multivariate logistic regression analysis

**Variables included in basic model**	**Beta (standard error)**	**OR (95% CI)**	**P**
Constant	18.922 (6.978)		
Age (years)	0.119 (0.025)	1.1 (1.1 - 1.2)	< 0.001
HDL-cholesterol (mmol/L)	– 1.165 (0.594)	0.3 (0.1 - 1.0)	0.050
Dominant HDL particle diameter (nm)	– 0.720 (0.271)	0.5 (0.3 - 0.8)	0.008
Dominant LDL particle diameter (nm)	– 0.716 (0.224)	0.5 (0.3 - 0.8)	0.001
sdLDL (%)	– 0.114 (0.041)	0.9 (0.8 - 1.0)	0.006
LDL III (%)	0.293 (0.082)	1.3 (1.1 - 1.6)	< 0.001
**Variables included in model A**			
Constant	13.141 (4.049)		
Age (years), (x_1_)	0.132 (0.025)	1.1 (1.1 - 1.2)	< 0.001
HDL-cholesterol (mmol/L), (x_2_)	– 1.26 (0.544)	0.3 (0.1 - 0.8)	0.021
Dominant HDL particle diameter (nm), (x_3_)	– 0.393 (0.231)	0.7 (0.4 - 1.1)	0.089
Dominant LDL particle diameter (nm), (x_4_)	– 0.612 (0.165)	0.5 (0.4 - 0.7)	< 0.001
**Variables included in model B**			
Constant	– 4.187 (2.694)		
Age (years), (x_1_)	0.14 (0.025)	1.2 (1.1 - 1.2)	< 0.001
HDL-cholesterol (mmol/L), (x_2_)	– 1.634 (0.521)	0.2 (0.1 - 0.5)	0.002
Dominant HDL particle diameter (nm), (x_3_)	– 0.51 (0.22)	0.6 (0.4 - 0.9)	0.020
sdLDL (%),(x_4_)	0.05 (0.02)	1.1 (1.0 - 1.1)	0.012
HDL - high-density lipoprotein. sdLDL - small, dense LDL particles. OR - odds ratio. CI - confidence interval. Basic model includes: age, HDL-cholesterol, dominant HDL and LDL particle diameters, relative proportions of sdLDL and LDL III subclasses. Model A includes: age, HDL-cholesterol, dominant HDL and LDL particle diameters. Model B includes: age, HDL-cholesterol, dominant HDL particle diameters and relative proportions of sdLDL. x_1_-x_4_ are variables to be entered in cost-effective models for calculation of expected probabilities of CRC development. Beta values represent regression coefficients to be entered in cost-effective models for calculation of expected probabilities of CRC development.			

First cost-effective model (Table 4) was formed by following variables: age, HDL-cholesterol, dominant HDL and LDL particle diameters. Hosmer-Lemeshow goodness of fit was χ^2^ = 7.761, d.f. = 8, P = 0.457 with Nagelkerke R^2^ value of 0.547 and 81% of correct patient classification. AUC was 0.89 (0.84 - 0.94). A model with relative proportion of sdLDL instead of dominant LDL particle diameter had weaker capability for correct patient classification (75%) and lower accuracy [AUC = 0.86 (0.81 - 0.92)] compared to first model. Hosmer-Lemeshow goodness of fit (χ^2^ = 10.089, d.f. = 8, P = 0.259) was adequate and Nagelkerke R^2^ value was 0.503. According to Hosmer-Lemeshow goodness of fit (χ^2^ = 16.492, d.f. = 8, P = 0.036) variables: age, HDL-cholesterol, dominant HDL particle diameter and relative proportion of LDL III subclasses did not fit well (data not shown). Regression coefficients for each cost-effective model that could be used for calculation of expected probabilities of CRC development are presented in the Table 4.

## Discussion

The present study constitutes the first report about LDL and HDL subclasses ability to predict the risk of CRC development. Also, this study is among a few researches that explored lipoprotein subclasses characteristics in cancer generally.

In the current study, we found that the concentrations of total cholesterol, LDL-cholesterol and HDL-cholesterol are decreased in CRC patients when compared to the controls. In addition, our results suggest that the decreased HDL-cholesterol has an independent potential for prediction of CRC development. Previously, it has been shown that malignant cells can accumulate cholesterol (*12*). Hence, it is possible that such redistribution of cholesterol in favour of cancerous cells cause diminishing of its entire amount in plasma, regardless of its lipoprotein carrier. This assumption also leads to the conclusion that the assessment of routine lipid profile does not provide sufficient information on possible roles of particular lipoproteins in development of CRC.

We found a shift toward smaller LDL particles in CRC patients in spite of lower LDL-cholesterol concentration in the same group. This finding suggests that determination of LDL particle diameter or proportion of sdLDL, rather than total LDL-cholesterol concentration in blood, could have higher significance in assessing the risk of CRC development. In addition, we confirmed that even if decreased LDL-cholesterol concentration was significant predictor of CRC, only smaller dominant LDL particle diameter, increased proportions of sdLDL particles and increased proportion of LDL III subclasses had independent power for prediction of the disease development. Our findings challenge the traditional interpretation of the mechanism for sdLDL subclasses formation. A possible explanation for simultaneous presence of elevated proportions of sdLDL particles and decreased concentrations of LDL-cholesterol could be found in the fact that cancer cells have greater requirement for cholesterol than normal cells. Namely, it is known that cancer cells overexpress LDL receptors, and as a consequence, LDL particles are being intensively absorbed by cancer cells. Since sdLDL particles own a lower affinity for LDL receptors they persist longer in plasma. Besides that, sdLDL particles are more susceptible to oxidation than larger LDL particles, thus creating oxidized LDL (oxLDL) that could be involved in the development of cancer. It has been shown that higher concentration of oxLDL may contribute to CRC development, probably by binding to oxidized LDL receptor (OLR1) that significantly contributes to the transformation, cell motility and growth of cancer cell lines (*13*). The potential significance of OLR1 in tumorigenesis was also observed in the study conducted by Khaidakov *et al.* who found that OLR1 has pro-oncogenic characteristics that are achieved through the activation of nuclear factor-κB (NF-kB) signalling pathway and genes responsible for de novo lipogenesis (*14*).

It is well known that HDL subclasses are not equally capable of accomplishing their protective function and that particular subspecies even lack protective properties (*15*, *16*). In our present study, we found significantly smaller dominant HDL particle diameter, increased proportion of HDL 3b and reduced proportion of larger HDL 2b particles in patients when compared to the controls. Our current results are consistent with those reported by Michalaki *et al.* in a study conducted in postmenopausal patients with breast cancer (*17*). Namely, previous studies have shown that even if small, dense HDL particles display essential protective properties, their activities could be compromised due to alterations in physicochemical characteristics in conditions accompanied with inflammation, oxidative stress and dyslipidemia (*16*). Additionally, the possible explanation for this discrepancy could be found in the fact that some types of cancer cells, especially those highly proliferative, accumulate cholesterol as cholesterol-esters in lipid droplets, thereby causing a decreased efflux of cholesterol to smaller HDL particles (*12*, *18*). Hence smaller HDL particles do not mature to larger HDL particles and they accumulate in plasma instead.

Compared to relative proportions of sdLDL and LDL III subclasses, dominant LDL particle diameter was superior in CRC prediction after the addition of age, HDL-cholesterol and dominant HDL particle diameter. This finding has important practical consequences, since dominant LDL and HDL particle diameters can be assessed by all major separation techniques, except by ultracentrifugation. Currently available methods employ various physicochemical properties of lipoproteins in order to separate their subclasses. For instance, the Vertical Auto Profile-II (VAP-II) ultracentrifugation method measures cholesterol content of the main lipoprotein subclasses. It is relatively fast and more practical for analysis of routine specimens, but limited data are available regarding comparison of VAP-II to other techniques. The most recently developed ion mobility method provides data on lipoprotein size and particle concentration (number) and has a high-throughput, similarly as the NMR methodology. Nevertheless, gradient gel electrophoresis remains the most used technique for lipoprotein subclasses characterisation, either as clinically available or in-house developed method, even if it is labor-intensive and time-consuming (*19*, *20*).

It was surprising to see the dominance of lipid parameters in terms of prediction accuracy when compared to gender, BMI, and glucose concentration. There is a strong evidence for direct association between obesity and the risk of various types of cancer. The altered metabolism of hormones, especially insulin, insulin-like growth factors and sex steroids, as well as the disturbed secretion of adipokines, have been proposed as plausible mechanisms that may encourage or promote cancer occurrence or progression in obese individuals (*21*). A meta-analysis of 31 studies have reported that for every 2 cm increment in waist circumstances and for every 2 kg/m^2^ increment in BMI, the risk for CRC development increased by 4% and 7%, respectively (*22*). However, in our study no difference was found in BMI between CRC patients and controls. Furthermore, we observed a trend towards lower BMI values in our patients. A possible explanation for the observed result might lie in the fact that for a significant number of our patients, the disease was classified as advanced one at the moment when they first came to the medical institution. It is possible that the prolonged disease duration caused malnutrition, which is typical for advanced stages of CRC, and therefore we failed to observe the link between obesity and CRC in our cohort. Malnutrition and cachexia seriously affect the quality of life of cancer patients and contribute to poor survival and to inadequate response to the prescribed therapy (*23*). Both are characterized by reduced adiposity that arise from increased lipolysis, rather than reduced lipogenesis (*24*). Furthermore, an altered lipid metabolism is closely related to cancer cell growth, proliferation, differentiation and, consequently, to cancer spread. Thus, it is clear that routine lipid profiling or determination of solely quantitative changes of blood cholesterol might not be sufficiently illustrative for dyslipidemia in CRC. Profound disturbances were seen only upon inclusion of quantitative characteristics of serum lipoproteins. These findings highlight the possible benefit of advanced lipid testing in specific metabolic conditions such as cancer-related malnutrition and consequent dyslipidemia.

For the first time lipid markers, LDL and HDL particle diameters and proportions of subclasses have been compared in terms of discriminative ability of CRC development in this study. Using the Hong Kong Diabetes Registry, Yang and colleagues have developed risk scores to predict all-cancer risk (*25*). Overall cancer risk score in that study had an AUC of 0.71, which is higher than the value obtained for a breast cancer risk scores (AUC = 0.66) proposed by Tice *et al.* (*26*). Compared to AUC for other diseases and cancer types, accuracies of our models had the most powerful values. Future cost-effectiveness analyses and clinical guidelines involving CRC screening should focus on individual patient risk probabilities calculation that includes LDL and HDL diameters, rather than on simple categorization of patients according to age, gender, BMI and serum lipids concentrations. These cost-effectiveness analyses could help to define biomarker profiles at which CRC screening becomes cost-effective.

Apart from the role of advanced lipid testing in stratification of risk and prevention of CRC development, the usefulness of these analyses with respect to treatment modality and progression of the disease is worth of investigation. Previous researches pointed towards the qualitative lipoprotein analysis in determining the optimal therapeutic approach in atherosclerosis-related diseases (*27*). Whether such approach might be useful in treatment of CRC remains to be established. In addition, it is noteworthy that several studies have hypothesized that use of statins may reduce the risk of CRC. Some of them reported that use of regular dose of statins did not appear to be associated with reduced risk of CRC development, while other showed that the long-term use of high doses of statins is associated with a lower incidence of distant metastases and a better clinical outcome (*28*, *29*). Future studies are needed to evaluate whether determination of lipoprotein subclasses might be useful in assessing the effectiveness of statins in preventing the onset and further progression of CRC. The prognostic value of LDL-cholesterol and HDL-cholesterol in metastatic CRC were reported by Liao *et al.* who found that increased LDL-cholesterol is independently associated with poor survival (*30*). To best of our knowledge, no previous studies analysed LDL and HDL subclasses distribution in metastatic CRC patients. In our study, only 9 patients had diagnosed metastatic changes, which was insufficient for relevant statistical analysis. However, this topic should be further explored in future studies.

Several drawbacks should be mentioned. First, we focused solely on the use of parameters of advanced lipid profile for assessment of CRC risk and designing cost-effective models, while we did not perform comparison with other cancer markers which are already in clinical use. Second, cross-sectional nature of our research did not allow us to validate the constructed models in prospective analysis. Finally, the patients and controls were not matched by age, due to difficulties to comply with the strict exclusion criteria for the control group in older population. However, an age-matched control group would provide additional strength to our conclusions. Future prospective studies are needed to verify our preliminary findings.

In conclusion, our results have demonstrated for the first time that patients with CRC have decreased LDL and HDL particle diameters and that the subclasses distribution is shifted towards smaller particles. Smaller HDL and LDL particle diameters were recognized as independent predictors of CRC. These two parameters of advanced lipid profile, alongside with age and HDL-cholesterol concentrations formed the optimal cost-effective model with adequate discriminative abilities. In spite of the confirmed clinical significance, determination of LDL and HDL particle diameters is rarely performed routinely, while routine assessment of LDL and HDL subclasses is not recommended at this moment. However, this study could provide a ground for further research concerning significance of qualitative lipoprotein analysis in the CRC risk evaluation.
